# Efficacy and safety of anti-tau drugs for Alzheimer’s disease: A systematic review and network meta-analysis

**DOI:** 10.1016/j.tjpad.2026.100693

**Published:** 2026-11-07

**Authors:** Yuetao Hu, Yuning Cheng, Yanqu Su, Zeming Han, Xiaohan Shen, Hantian Hu, Qi Wang, Siqi Wan, Hanyu Li, Nan Zhang

**Affiliations:** Department of Neurology, Tianjin Neurological Institute, Tianjin Medical University General Hospital, 154 Anshan Road, Tianjin, 300052, China

**Keywords:** Alzheimer’s disease, Anti-tau drugs, Network meta-analysis, Meta-regression

## Abstract

**Objective:**

To evaluate and compare the effects of different anti-tau drugs on clinical and biological outcomes in patients with Alzheimer’s disease (AD).

**Methods:**

PubMed, Cochrane Library, and Embase databases were searched for identifying randomized, controlled clinical trials from inception to December 2025.

**Results:**

Twelve high-quality studies with 11 drugs were evaluated. Systematic meta-analysis did not demonstrate significant efficacy of anti-tau drugs in preventing cognitive and functional declines or clearance of tau deposition on PET. However, effects in reducing levels of p-tau (SMD=-0.48, 95% CI: -0.73 to -0.22, P < 0.01) and t-tau (SMD=-0.69, 95% CI: -0.94 to -0.45, P < 0.01) in the cerebrospinal fluid were observed. Network analysis revealed that MAPT_Rx_ and nicotinamide ranked highest for improving cognitive function, and gosuranemab ranked highest for tau biomarker changes.

**Conclusion:**

The current findings highlight the disease-modifying potential of anti-tau therapies for AD, although clinical benefit is limited. The observed reductions in CSF tau biomarkers and the favourable safety profile of current anti-tau therapies suggest promising therapeutic potential for the treatment of AD. However, the clinical value of anti-tau therapies should be confirmed in adequately powered phase III trials with biomarker-enriched populations.

## Highlights

• Anti-tau therapies significantly reduce cerebrospinal fluid p-tau and t-tau levels.
• Anti-tau therapies do not significantly improve cognitive and functional outcomes.
• MAPT_Rx_ and nicotinamide improve cognitive function with the greatest efficacy.
• Meta-regression shows age is associated with t-tau reduction post anti-tau therapy.
• Anti-tau drugs are safe, except for a higher risk of injection-site reactions.

## 1 Background

Alzheimer’s disease (AD) is the most common form of neurodegenerative dementia and one of the top five leading causes of death worldwide [1]. In recent decades, the treatment of AD has focused on alleviating cognitive symptoms but has failed to halt disease progression [2]. Commonly used drugs, including cholinesterase inhibitors and memantine, have proven efficacy in symptom relief [3]. Recently, anti-amyloid-β (Aβ) therapy, particularly monoclonal antibodies targeting Aβ [4], e.g., lecanemab (Leqembi) and donanemab (Kisunla), has been shown to significantly slow disease progression and has been approved for the treatment of early-stage AD patients [5], including those with mild cognitive impairment (MCI) due to AD and mild AD dementia. However, this anti-Aβ therapy still has only limited efficacy in delaying the clinical progression of AD and does not significantly affect patients at advanced stages [6].

Notably, anti-tau therapy, whether as a monotherapy or in combination with anti-Aβ therapy, is considered a promising therapeutic approach [7]. Tau is a microtubule-associated protein predominantly localized to the neuronal axons, where it orchestrates microtubule stabilization and axonal transport through dynamic tubulin interactions under physiological conditions [8]. Instead of binding to microtubules, soluble tau proteins undergo various modifications under pathological conditions [9], such as hyperphosphorylation. The accumulation of phosphorylated tau proteins subsequently leads to the formation of neurofibrillary tangles, contributing to synaptic damage, neuronal dysfunction, and ultimately cognitive decline [10].

Current tau protein-targeting therapeutic strategies have significant pathological implications, such as reducing tau expression, targeting tau protein modifications, inhibiting tau aggregation and stabilizing microtubules, but these strategies still face bottlenecks in clinical translation. Among novel drugs, the anti-tau vaccine AADvac1 demonstrated therapeutic promise in a phase II clinical trial, with a seroconversion rate of 98.2% and slowing of the expected increase in plasma neurofilament light chain (NfL) levels (12.6% change from baseline vs. 27.7% in placebo). Post hoc analysis revealed that AADvac1 reduced the rate of accumulation of plasma NfL by 56% in plasma phosphorylated tau (p-tau)217-positive patients [11,12]. Similarly, in phase Ib trials, the tau-targeting antisense oligonucleotide MAPT_Rx_ resulted in a 51% reduction in total tau protein (t-tau) levels in the cerebrospinal fluid (CSF) in the high-dose treatment group [2]. However, the clinical efficacy and safety of tau-targeting therapies require additional supporting evidence. While the GSK-3β inhibitor tideglusib reduced CSF t-tau levels, it did not significantly affect cognitive function (Alzheimer’s Disease Cooperative Study-Activities of Daily Living (ADCS-ADL) change: −2.6 for treatment vs. −2.2 for placebo, *P* > 0.05) [13]. Similarly, LMTM, a selective inhibitor of tau protein aggregation, did not significantly improve cognitive or functional outcomes (Alzheimer’s Disease Assessment Scale-Cognitive Subscale (ADAS-Cog) and ADCS-ADL; *P* > 0.86) in mild to moderate AD and was associated with increased rates of gastrointestinal and urinary adverse events, causing increased discontinuation (9% treatment vs. 2% control) [14].

Given the limited evidence for the efficacy and safety of these drugs, it is necessary to comprehensively summarize the results of previous and current clinical trials of candidate anti-tau drugs and define the boundaries of current evidence: specifically, which fluid biomarkers (e.g., CSF t-tau and p-tau) and imaging biomarkers (e.g., tau PET) are treatment-responsive, and which clinical outcomes (e.g., cognitive and daily function) are not. These evidence boundaries can inform future trial design.

## 2 Methods

### 2.1 Search strategy

This study was registered on PROSPERO (CRD42025647894) and followed the Preferred Reporting Items for Systematic Reviews and Meta-Analysis (PRISMA) statement and the Cochrane Handbook guidelines for meta-analysis [15,16].

We searched PubMed, the Cochrane Library, and Embase from inception to December 2025 with the following search terms: “Alzheimer’s disease”, “tau OR tau protein OR t-tau OR p-tau OR ttau OR ptau OR neurofibrillary tangle OR microtubule-associated proteins tau”, “therapy OR treatment”, and “antibody OR drug OR vaccine”. Two reviewers (CY and SY) removed duplicate records and screened titles and abstracts and then full-text manuscripts according to predetermined inclusion and exclusion criteria using EndNote X9/Zotero 7.0 software independently. Disagreements were resolved by discussion or, if necessary, by third-party adjudication.

### 2.2 Inclusion and exclusion criteria

To be included in the meta-analysis, a study had to (1) be a Phase IB/II/III parallel-design, double-blind, randomized, placebo-controlled trials (RCTs), evaluating the efficacy and safety of tau-targeting antibodies, vaccines, or small molecular drugs; (2) have medium or low risk of bias tested using Review Manager; (3) include participants with sporadic AD dementia at any stage; (4) report at least one primary clinical efficacy outcome, including cognitive function and/or daily function, assessed using one or more of the following measures: Alzheimer’s Disease Assessment Scale-Cognitive Subscale (ADAS-Cog), Clinical Dementia Rating scale-Sum of Boxes (CDR-SB), AD Cooperative Study’s Activities of Daily Living (ADCS-ADL), Mini Mental State Examination (MMSE) and (5) be published in any language.

Studies were excluded if they (1) were narrative/systematic reviews, meta-analyses, phase IA RCTs, single-arm trials, prospective cohort studies, case–control studies, cross-sectional studies, case reports, opinions/editorials, or post hoc/secondary analyses of primary studies; (2) had a high or unclear risk of bias according to the ROB tool; (3) involved hereditary AD or other types of dementia; (4) did not report cognitive/functional outcomes; or (5) were nonhuman studies.

### 2.3 Data extraction and quality assessment

For each eligible study, the following information was extracted: publication information (year of publication, study duration, and author), study design (including sample size, treatment and follow-up period, randomization, and blinding), demographic information (age, sex, and education level), interventions (drug, dosage), and outcomes.

We assessed the risk of bias in the included trials using the ROB tool (version 2.0) [17]. The assessment covered the following domains: selection bias, allocation concealment, performance bias, reporting bias, and other biases. Each domain was recorded as "yes," "no," or "unclear," where "yes" indicated a low risk of bias, "no" indicated a high risk of bias, and "unclear" indicated insufficient information or unknown risk of bias. Disagreements were resolved by discussion or, if necessary, by third-party adjudication.

### 2.4 Data analysis

Multiple measures were used in this study to evaluate the effectiveness and safety of anti-tau treatments for AD. We categorized the included data into primary outcomes demonstrating clinical efficacy, secondary outcomes exploring biomarker changes, and adverse events demonstrating issues with safety. Specifically, the primary outcomes included ADAS-Cog, CDR-SB, ADCS-ADL and MMSE; the secondary outcomes included CSF t-tau, p-tau, and the tau PET composite standardized uptake value ratio (SUVR); and the safety outcomes included fatigue, nausea, diarrhea, dizziness, falls, urinary tract infections and injection-site reactions. Outcomes were included in the meta-analysis only when reported by at least three study arms.

We first performed random-effects pairwise meta-analyses for each outcome and compared the intervention group with the placebo group using the metan module in Stata software, version 15.0. For continuous variables (including ADAS-Cog, CDR-SB, ADCS-ADL and MMSE scores, CSF t-tau and p-tau concentrations, and tau PET SUVR), we calculated standardized mean differences (SMDs) using Hedges’g with 95% confidence intervals (CIs). For categorical variables (e.g., incidence rate of adverse events), we computed log risk ratios (LogRRs) with 95% CIs. When studies included multiple interventions or detected arms (e.g., different doses, different brain regions of tau PET), all the relevant experimental arms of the study were combined into a single group, and the means and standard deviations (SDs) were merged using formulas for continuous outcomes in the Cochrane Handbook’s formula for continuous outcomes mainly for the purpose of unifying inclusion criteria, reducing statistical dispersion and ensuring adequate pooled sample size for overall effect evaluation [16,18], while the total number of events and the total number of participants from all relevant arms were pooled for the calculation of the risk ratio (RR) for dichotomous outcomes. I² statistics were calculated (low heterogeneity: <0.25; moderate heterogeneity: 0.25–0.75; high heterogeneity: >0.75) to assess heterogeneity in the pooled effect estimates. When significant heterogeneity was observed (I²>0.50), a random-effects model was employed; otherwise, a fixed-effects model was used. An outlier and influence analysis was conducted to identify potential outliers and influential observations [19]. Studentized deleted residuals (SDRs) were calculated to detect outliers, with observations identified as outliers if the absolute SDR value exceeded 1.96 [19,20]. Cook’s distance (CD) was used to assess the influence of individual effect sizes, and effect sizes with CD values exceeding 0.45 were considered influential [21]_._ To ensure the validity and robustness of the results, influential outliers were excluded from the final dataset [19,22]. Begg’s test and Egger’s test were employed to assess the robustness of the results, and sensitivity analyses were performed using the leave-one-out approach.

We further performed network meta-analyses using the Markov chain-Monte Carlo (MCMC) simulation technique and implemented the network within a frequentist framework [23]. Four chains were run with 10,000 simulation iterations, 2500 adaptation iterations, and a thinning interval of 10; convergence was assessed via trace plots, density plots, and Brooks–Gelman–Rubin diagnostic plots. We performed indirect comparisons because there was no head-to-head comparison between drugs in any of the included clinical trials. We used the node-splitting approach to evaluate inconsistencies between direct and indirect estimates within each closed loop and applied a design-by-treatment interaction model to assess the entire network [24]. Furthermore, we created network plots using Stata software, version 16.0, and ranked drug effects on cognitive/functional changes using the surface under the cumulative ranking curve (SUCRA) to evaluate treatment efficacy [25]. SUCRA values are expressed as percentages, where 100% indicates a treatment certain to be the best and 0% certain to be the worst. Network diagrams were generated to visualize comparisons among all the available drugs and placebos in the included trials.

Additionally, to assess whether baseline characteristics influenced outcomes, we performed meta-regression to assess correlations between drug efficacy (the SMDs with 95% CIs of primary outcomes and secondary outcomes) and demographic, clinical, and biological status at baseline, including age, sex, clinical severity (assessed by MMSE score and CDR-SB score), and biological severity (assessed by t-tau, p-tau and tau PET SUVR). Furthermore, the association between efficacy and tau reduction was analyzed using meta-regression by estimating the weighted mean differences (MDs) with 95% CIs using an inverse-variance random-effects model [26].

## 3 Results

Our literature search identified 361 publications. After excluding duplicates (n = 146) and studies that did not meet the inclusion criteria (n = 200), 15 RCTs were included (Fig. 1). These trials covered phase IB (n = 2), phase II (n = 12), and phase III (n = 1) clinical studies, all of which utilized tau-targeting therapies in the treatment groups and included placebo controls. Treatment durations ranged from 10 to 104 weeks. The sample sizes ranged from 40 to 650 participants, encompassing a total of 3093 patients of diverse ethnic backgrounds worldwide. The mean age was 70.73 years old (SD = 7.44) in the treatment groups and 71.28 years old (SD = 7.65) in the control groups. Female participants composed 55.20% of the treatment groups and 53.04% of the control groups. The mean years of education were 14.24 (SD = 3.73) in the treatment groups and 14.39 years (SD = 3.64) in the control groups, with 8 studies (66.67%) lacking education data.

**Fig. 1:**
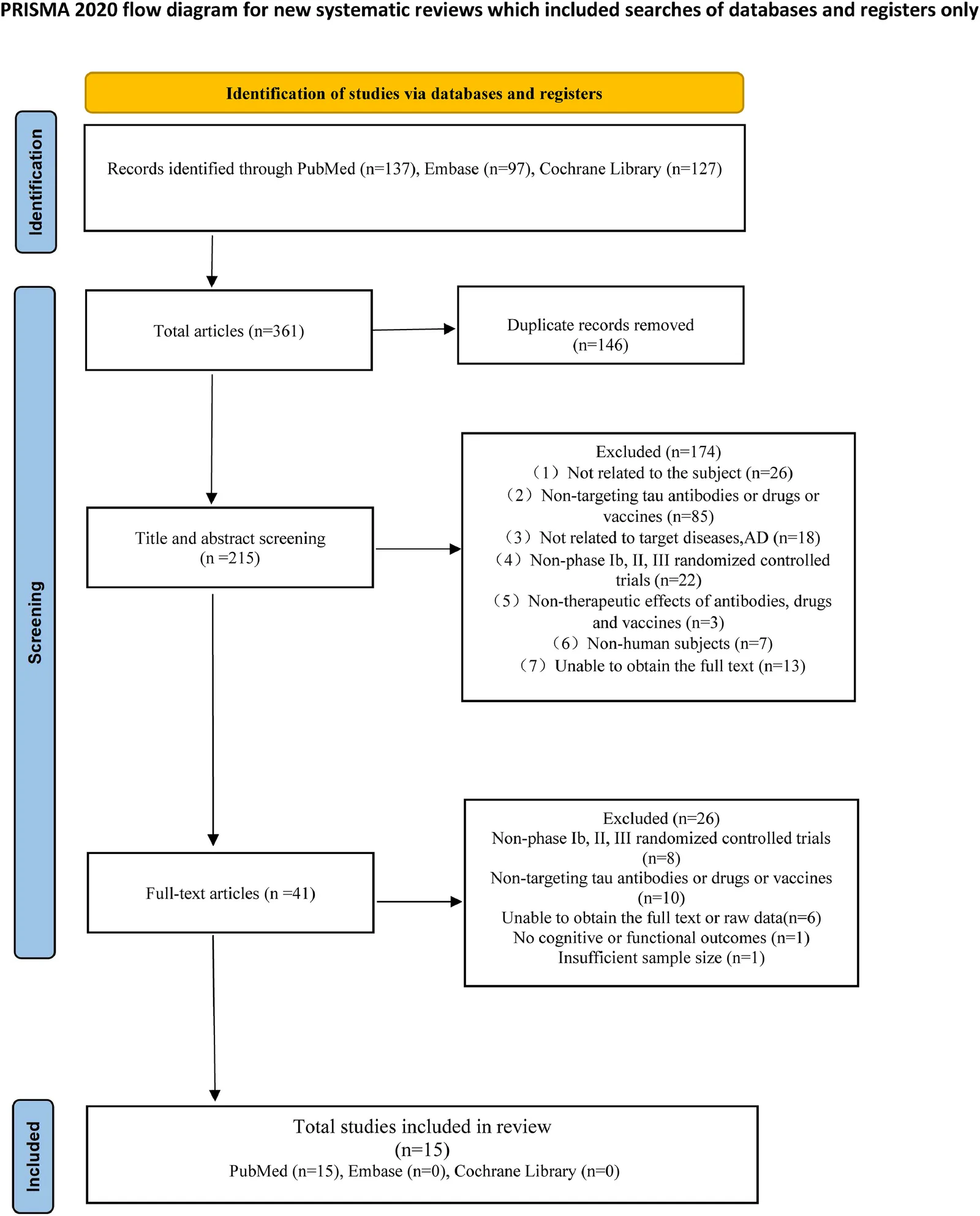
Flow chart of the study selection process.

We assessed the risk of bias in the 15 included studies. All the studies had low risks of bias in terms of allocation concealment, the blinding of the participants and personnel, the blinding of outcome assessments and selective reporting. One study showed high other bias due to COVID-19-related disruptions, including limited longitudinal CSF samples and insufficient racial/ethnic diversity, but sensitivity analyses yielded results similar to those of the overall analyses; thus, this study was still included in the final analysis (appendix 1). However, 3 studies were excluded from our study because they did not meet the inclusion criteria, and their original data could not be obtained: for LMTM (Serge Gauthier et al. [14]), cognitive or functional outcomes were reported without numerical comparisons with placebo, and fluid biomarker outcomes were not presented; for lithium (Marielza Andrade Nunes et al. [27]), cognitive or functional outcomes were presented as figures without extractable numerical values; and for ACI-35 (Olivier Sol et al. [28]), the published report did not include the required cognitive or functional outcomes. Finally, 12 studies with good overall quality were included.

Ultimately, the following 11 anti-tau agents were assessed: 4 direct anti-tau monoclonal antibodies, namely zagotenemab, tilavonemab, gosuranemab, semorinemab; 1 active immunotherapy vaccine, AADvac1; 1 tau aggregation inhibitor, MT; 4 tau phosphorylation modulators, including lithium, nicotinamide, VEL015, and tideglusib; and 1 tau-targeting antisense oligonucleotide, MAPT_Rx_ (Table 1).

**Table 1:** Characteristics of included clinical trials.

**Study**	**Drug**	**Year of publication /duration(weeks)**	**No. of patients (treatment/placebo)**	**Age (Treatment/Placebo) (Mean (SD))**	**Sex (Female) (Treatment/Placebo)**	**Education, years**	**Clinical stage**	**Baseline MMSE (treatment/ placebo)**	**Baseline ADAS-Cog (treatment/ placebo)**	**Primary Efficacy outcome**	**Neuroimaging / Biomarker outcomes**	**Baseline t-tau**	**Baseline p-tau**	**Baseline tau PET SUVR**
**Wischik, C. M.**	MT	2015/24	229/92	73.5(9.4)/74.6(8.1)	127/45	NR	Mild to moderate AD	19.2/19.6	25.0/24.5	ADAS-Cog	MTC,rCBF	NR	NR	NR
**Malpas, C. B.**	VEL015	2016/24	20/20	70/71	9/8	NR	Mild to moderate AD	1	22.08/19.68	MMSE	PET,MRI	1028(481.42)/884(346.81)	92 (38.79) /81(27.38)	1.14(0.205)/1.12(0.319)
**Petr Novak**	AADvac1	2021/104	117/79	70.5(8.33)/72.7(6.86)	61/45	12.9(3.49)/12.2(3.33)	Mild AD	23.0(1.90)/23.2(1.95)	NR	CDR-SB,ADCS-ADL	NfL,t-tau,p-tau T181,hippocampal volume, entorhinal cortex volume, whole brain volume	NR	NR	NR
**Edmond Teng**	Semorinemab	2022/73	296/126	69.5(6.9)/69.8(7.3)	164/71	NR	Prodromal to mild AD	23.4(2.6)/23.1(2.6)	27.5(7.1)/28.7(8.1)	CDR-SB, ADAS-Cog, MMSE, ADCS-ADL	t-tau, p-tau, tau PET	379.60(203.90)/331.6 (140.3)	NR	1.33(0.25)/1.34(0.26)
**Hana Florian**	Tilavonemab	2023/96	337/116	71.2 (7.1) /71.7 (6.7)	164/70	14.7 (3.8) /14.9 (3.4)	MCI or probable AD	24.5/24.0	27.1/26.3/26.5/26.2	CDR-SB	MRI, Abeta PET, NFL, free tau and t-tau, plasma tau	NR	NR	NR
**Cecilia Monteiro**	Semorinemab	2023/96	123/115	71 (8.3) /73 (8.75)	84/70	NR	Mild to moderate AD	18.4/18.2	23.9/24.1	ADAS-Cog, ADCS-ADL	t-tau, p-tau, MRI, tau PET	NR	NR	1.4(0.3)/1.4(0.3)
**Catherine J Mummery**	MAPTRx	2023/72	34/12	66 (6.1) /66 (4.6)	17/6	NR	Mild AD	23.5/24.2	NR	CDR-SB, ADCS-ADL, MMSE, ADAS-Cog	t-tau, p-tau	405.6(132.7)/387.3(120.9)	NR	NR
**Melanie Shulman**	gosuranemab	2023/78	436/214	69.7 (6.9) /69.8 (6.6)	221/106	14.2 (3.7) /14.8 (3.7)	MCI due to AD or mild AD	25.3/25.4	25.6/26.2/27.1/25.3/26.4	CDR-SB, ADAS-Cog, MMSE, ADCS-ADL, FAQ	t-tau, p-tau, Aβ42, tau PET, MRI	NR	NR	1.855(0.705)/1.885(0.705)
**Adam S Fleisher**	Zagotenemab	2024/100	242/118	75.4/75.3	137/53	NR	MCI or mild dementia due to AD	23.84/23.51/23.65	28.08/29.63/28.78	CDR-SB, ADAS-Cog, MMSE, ADCS-ADL	t-tau, p-tau, vMRI, tau PET	NR	NR	1.230(0.114)/1.22(0.117)
**Harald Hampel**	lithium	2008/10	33/38	68.2(7.2)/68.9(8.3)	18/19	NR	Mild AD	23.6(1.6)/23.6(1.7)	15.8(4.2)/15.4(5)	ADAS-Cog,MMSE	t-tau, p-tau	721.72(326.29)/608.43(245.45)	NR	NR
**Lovestone**	tideglusib	2015/52	165/85	71.7(3.5)/70.8(8.6)	123/47	NR	Mild to moderate AD	19.6(4.1)/19.9(4.2)	29.8(9.2)/30.7(9.4)	ADAS-Cog,MMSE	MRI	NR	NR	NR
**Joshua D. Grill**	Nicotinamide	2025/48	24/22	74.6 (9) /72.9 (7.4)	10/10	15.2(2.5)/15.6(2.1)	MCI or mild dementia	25.6/25.5	24.7/24.2	CDR-SB, ADCS-ADL, MMSE, ADAS-Cog	t-tau, p-tau, MRI	667.7(436.1)/522.7(218.3)	NR	NR

NR indicates not reported or not retrievable. ADAS-Cog versions varied across studies (e.g., 11-, 13-, 14-, and 15-item versions), as did the tau PET tracers (e.g., [¹⁸F]GTP1, [¹⁸F]MK-6240, and [¹⁸F]flortaucipir).

Abbreviations: ADAS-Cog, Alzheimer’s Disease Assessment Scale-Cognitive Subscale; CDR-SB, Clinical Dementia Rating-Sum of Boxes; ADCS-ADL, Alzheimer’s Disease Cooperative Study-Activities of Daily Living; MMSE, Mini Mental State Examination; t-tau, total tau protein; p-tau, phosphorylated tau; SUVR, standardized uptake value ratio; PET, positron emission tomography; AD, Alzheimer’s disease; MCI, Mild Cognitive Impairment.

### 3.1 Influence analysis

To enhance the robustness of the findings, potential influential outliers were identified using outliers and influence diagnostics. In total, 3 studies appeared to be influential outliers. Specifically, in the primary outcome analyses, the study of nicotinamide (Joshua D. Grill et al. [29]) was identified as an outlier for the assessment of the CDR-SB (appendix 2.1). With respect to the secondary outcomes, the analysis of t-tau and p-tau levels revealed that MAPT_Rx_ (Catherine J Mummery et al. [2]) was an outlier (appendix 2.2). In the safety analyses, gosuranemab (Melanie Shulman et al. [30]) was identified as an outlier for injection-site reactions, whereas no outliers were detected for the other adverse events (appendix 2.3).

Studies that included outlier data were excluded from the corresponding analysis to ensure the validity of our results. A consistent analytical approach was adopted across all outcomes, wherein results obtained after the exclusion of outliers were reported alongside those derived from the complete dataset without such exclusions. The results obtained from all the studies (without outliers excluded) are provided in appendices 3, 4, 5 and 6.

### 3.2 Primary outcomes

Compared with placebo, no significant improvement was found in the ADAS-Cog [SMD=0.01 (95% CI: −0.08 to 0.10), I^2^=0.44, *P* = 0.85], CDR-SB [SMD=0.05 (95% CI: −0.05 to 0.16), I^2^<0.01, *P* = 0.33], ADCS-ADL [SMD=−0.09 (95% CI: −0.21 to 0.02), I^2^ =0.02, *P* = 0.12] and MMSE [SMD=−0.04 (95% CI: −0.14 to 0.07), I^2^=0.17, *P* = 0.50] scores (appendix 3.1.1). There was no evidence of publication bias, with Begg’s test p values of 0.28, 0.26, 0.22, and 0.08 and Egger’s test p values of 0.12, 0.47, 0.14, and 0.06, respectively (Table 2).

**Table 2:** Pooled estimations of clinical effects with additional Begg’s test and Egger’s test after outlier removal.

**Outcomes**	**SMD [95%CI]**	**τ**[2]	**P value**	**I**[2]	**H**[2]	**df**	**λ**[2]	**z**	**P for Cochran’s Q test**	**Begg’s test Kendall’s score**	**P for Begg’s test**	**Egger’s test t value**	**P for Egger’s test**
**ADAS-Cog**	−0.01[−0.08, 0.10]	-	0.850	43.80%	1.78	9	16.01	0.19	0.067	−13.00	0.245	−1.74	0.119
**CDR**	0.05[−0.05,0.16]	-	0.329	0.00%	0.11	5	0.53	0.98	0.992	−7.00	0.260	−0.79	0.472
**ADCS-ADL**	−0.09[−0.21,0.02]	-	0.122	1.80%	1.02	4	4.07	−1.55	0.396	−6.00	0.221	−2.02	0.136
**MMSE**	−0.04[−0.14,0.07]	-	0.495	17.40%	1.21	8	9.69	−0.68	0.287	18.00	0.076	2.22	0.062
**t-tau**	−0.69[−0.94, −0.45]	-	<0.01	39.70%	1.66	4	6.64	−5.48	0.156	2.00	0.806	1.37	0.263
**p-tau**	−0.48[−0.73, −0.22]	-	<0.01	0.00%	0.68	3	2.04	−3.71	0.563	2.00	0.734	0.29	0.798
**tau PET SUVR**	0.01[−0.12, 0.15]	-	0.862	0.00%	1.00	3	2.99	−2.19	0.393	4.00	0.308	2.26	0.152

Note: The **τ^2^** value is only reported for analyses using a random-effects model (I² ≥ 50%), while it is not applicable under the fixed-effects model (I² < 50%).

Abbreviations: ADAS-Cog, Alzheimer’s Disease Assessment Scale-Cognitive Subscale; CDR-SB, Clinical Dementia Rating-Sum of Boxes; ADCS-ADL, Alzheimer’s Disease Cooperative Study-Activities of Daily Living; MMSE, Mini Mental State Examination; t-tau, total tau protein in cerebrospinal fluid; p-tau, phosphorylated tau protein in cerebrospinal fluid; PET, positron emission tomography; SUVR standardized uptake value ratio.

The network plots for the primary outcome are illustrated in Fig. 2. Nicotinamide ranked highest for ADAS-Cog (SUCRA: 71.4), followed by lithium (SUCRA: 65.3). MAPT_Rx_ (SUCRA: 93.5), which ranked highest in MMSE, followed by semorinemab (SUCRA: 86.0). For ADCS-ADL and CDR-SB, all the interventions had lower SUCRA values than placebo did, indicating that none of the anti-tau drugs ranked higher than placebo did in terms of these outcomes (Table 3). The forest plot suggested that, compared with placebo, none of the treatments significantly improved all the primary outcomes.

**Fig. 2:**
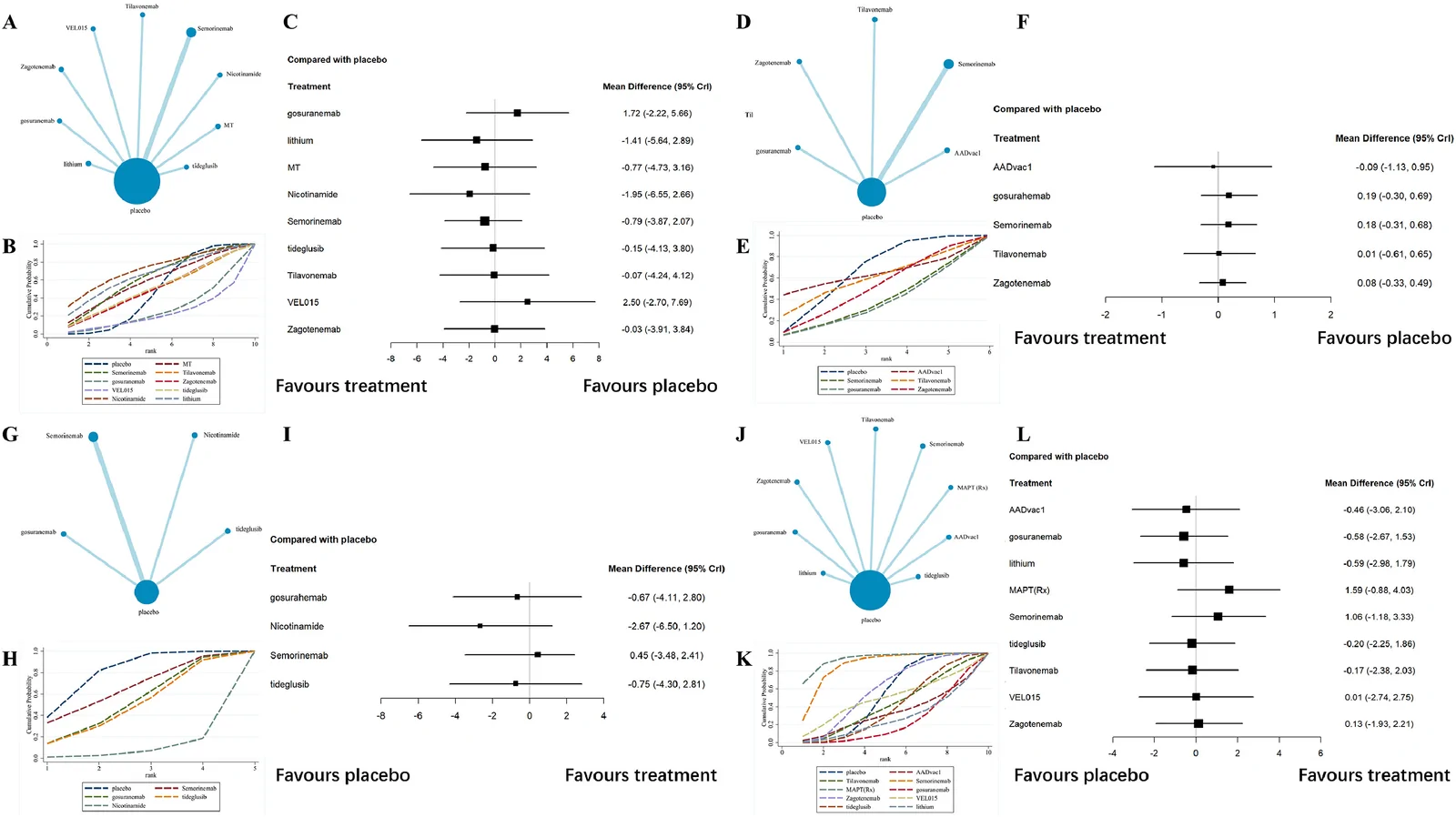
Network meta-analysis of the primary outcomes after outlier removal. For each cognitive or functional outcome, including ADAS-Cog (A-C), CDR-SB (D-F), ADCS-ADL (G-I), and MMSE (J-L), three plots are presented: (1) network plots show intervention comparisons, with node size reflecting participant numbers and edge thickness indicating sample sizes for direct comparisons (A, D, G, J); (2) SUCRA ranking plots illustrate the probability of each intervention being the best treatment (B, E, H, K); and (3) forest plots display network effect sizes vs. placebo with 95% credible intervals (Crl) (C, F, I, L).

Abbreviations: ADAS-Cog, Alzheimer’s Disease Assessment Scale-Cognitive Subscale; CDR-SB, Clinical Dementia Rating-Sum of Boxes; ADCS-ADL, Alzheimer’s Disease Cooperative Study-Activities of Daily Living; MMSE, Mini Mental State Examination; SUCRA, surface under the cumulative ranking curve.

**Table 3:** SUCRA values for all outcomes under different interventions after outlier removal.

Intervention	ADAS-Cog	ADCS-ADL	CDR-SB	MMSE	T-tau	P-tau	Tau PET SUVR
MT	58.4	-	-	-	-	-	-
Tilavonemab	49.3	-	57.3	41.4	-	-	-
Semorinemab	61.8	64.2	35.2	86.0	48.2	60.3	48.2
Gosuranemab	26.3	50.8	46.3	22.3	91.4	96.2	87.1
VEL015	21.6	-	-	48.7	40.9	33.9	-
Tideglusib	50.0	48.1	-	39.2	-	-	-
Nicotinamide	71.4	7.4	-	-	47.5	49.1	-
Lithium	65.3	-	-	26.0	-	-	-
AADvac1	-	-	61.8	32.4	61.9	-	-
Zagotenemab	-	-	48.4	58.4	-	-	10.4
MAPTRx	-	-	-	93.5	-	-	-
Placebo	46.8	79.5	63.9	52.2	10.0	10.5	54.4

Abbreviations: ADAS-Cog, Alzheimer’s Disease Assessment Scale-Cognitive Subscale; CDR-SB, Clinical Dementia Rating-Sum of Boxes; ADCS-ADL, Alzheimer’s Disease Cooperative Study-Activities of Daily Living; MMSE, Mini Mental State Examination; t-tau, total tau protein in cerebrospinal fluid; p-tau, phosphorylated tau protein in cerebrospinal fluid; PET, positron emission tomography; SUVR, standardized uptake value ratio.

### 3.3 Secondary outcomes

The results of the secondary outcomes are presented in appendix 3.1.2. Significant effects in reducing CSF p-tau levels [SMD=−0.48 (95% CI: −0.73 to −0.22), I^2^ <0.01, *P* < 0.01] and t-tau levels [SMD=−0.69 (95% CI: −0.94 to −0.45), I^2^=0.40, *P* < 0.01] were observed. No significant decrease was found in the tau PET composite SUVR [SMD=0.01 (95% CI: −0.12 to 0.15), I^2^ <0.01; *P* = 0.86]. There was no evidence of publication bias, with Begg’s test p values of 0.73, 0.81, and 0.31 and Egger’s test p values of 0.80, 0.26, and 0.15, respectively (Table 2).

The network plots for the secondary outcomes are illustrated in Fig. 3. The efficacy of various intervention measures was ranked as follows: gosuranemab ranked highest for t-tau (SUCRA: 91.4), p-tau (SUCRA: 96.2) and the tau PET composite SUVR (SUCRA: 87.1) (Table 3). The forest plot suggested that, compared with placebo, none of the treatments significantly improved any of the secondary outcomes.

**Fig. 3:**
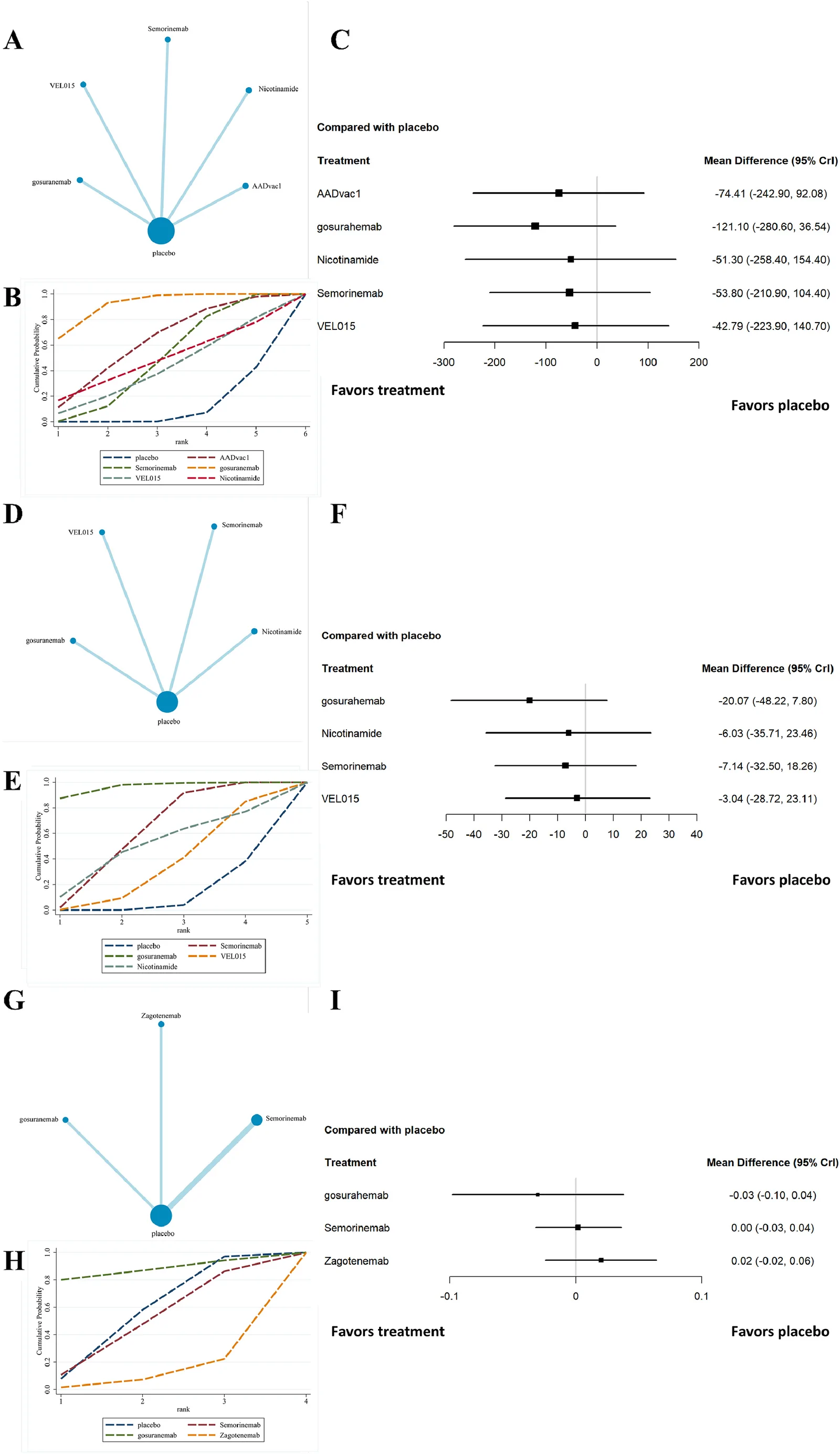
Network meta-analysis of secondary outcomes after outlier removal. For each biomarker outcome, including t-tau (A-C), p-tau (D-F), and tau PET composite SUVR (G-I), three plots are presented: (1) network plots show intervention comparisons, with node size reflecting participant numbers and edge thickness indicating sample sizes for direct comparisons (A, D, G); (2) SUCRA ranking plots illustrate the probability of each intervention being the best treatment (B, E, H); and (3) forest plots display network effect sizes vs. placebo with 95% credible intervals (C, F, I).

Abbreviations: t-tau, total tau protein in cerebrospinal fluid; p-tau, phosphorylated tau protein in cerebrospinal fluid. PET, positron emission tomography; SUVR, standardized uptake value ratio

### 3.4 Adverse events

The LogRRs of fatigue, nausea, diarrhea, dizziness, falls, urinary tract infections, and injection-site reactions were calculated as safety indices. After removal of outliers, injection-site reactions [logRR =1.19 (95% CI: 0.19 to 2.20), I^2^ <0.01, *P* < 0.05] were more frequent with the anti-tau treatments than with placebo treatment, but significant differences were not observed for other adverse events (Fig. 4). The results prior to outlier exclusion are provided in appendix 3.2.3.

**Fig. 4:**
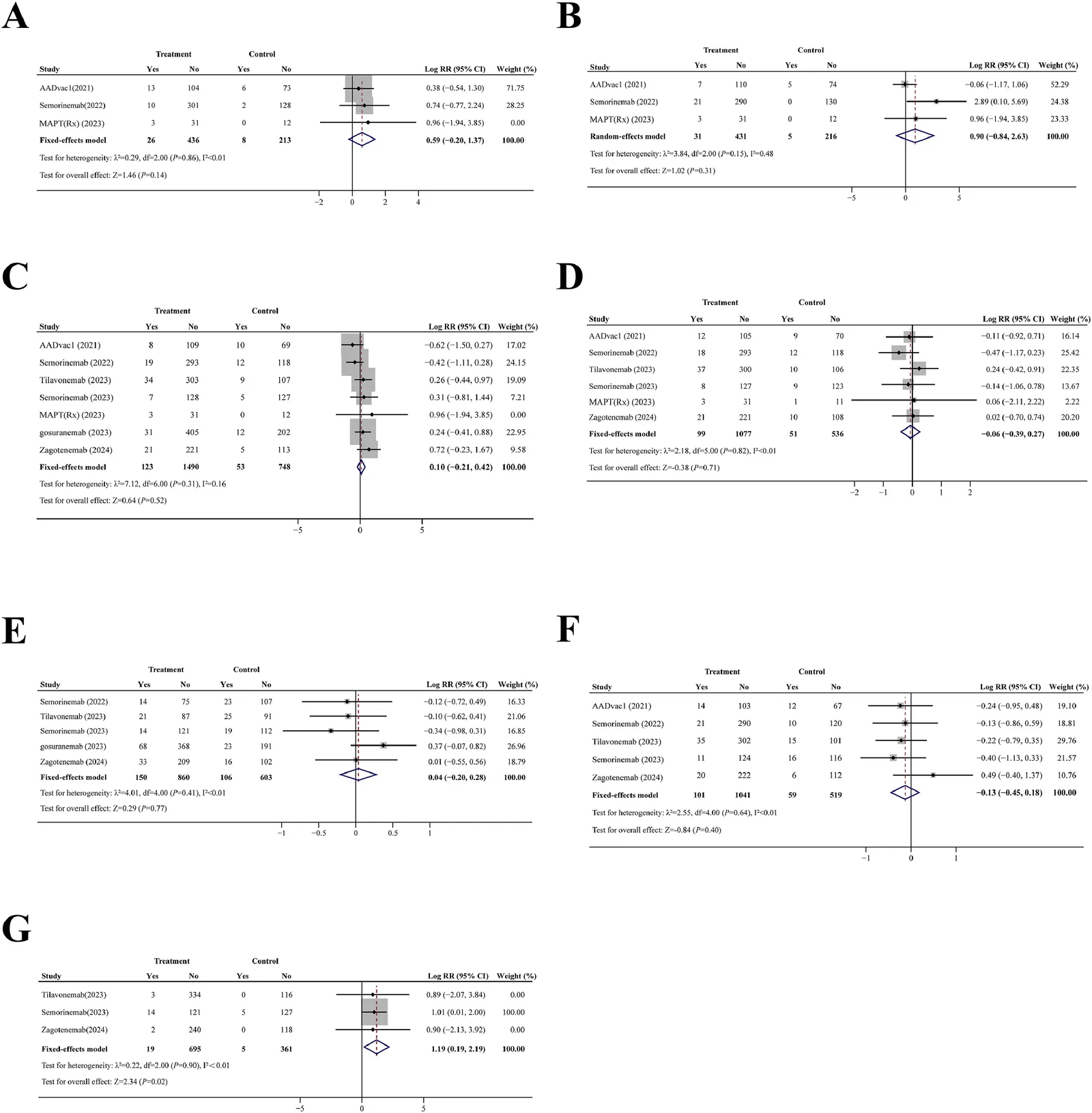
Forest plots of meta-analyses of adverse events after outlier removal. Effect sizes are presented as relative ratios with 95% confidence intervals (CIs). Forest plots depict meta-analysis results for adverse events of anti-tau drugs, including fatigue (A), nausea (B), diarrhea (C), dizziness (D), falls (E), urinary tract infections (F), and injection site reactions (G).

Abbreviations: CI, confidence interval.

### 3.5 Meta-regression

We performed meta-regression analyses to assess correlations between drug efficacy (primary outcomes and secondary outcomes) and baseline demographics and clinical and biological severity (appendix 5.1). A significant association between t-tau reduction and age was observed (β= 0.35; *P* = 0.039), but this association became nonsignificant after exclusion of the outlier (*P* = 0.268).

We further assessed the associations between efficacy and tau reduction, except those between both clinical outcomes (MMSE and CDR-SB) and p-tau reduction, after outlier removal because of limited data availability (appendix 5.1.3). No significant association was observed between any clinical outcome and the extent of tau reduction, either before or after the removal of outliers (all *P* > 0.05).

### 3.6 Sensitivity analyses

We performed sensitivity analyses on the primary and secondary outcomes and adverse event findings. Effect sizes remained relatively stable following the sequential exclusion of individual studies, indicating the robustness of these meta-analytic results. However, after removing the study of semorinemab, the increase in the risk of injection-site reactions was no longer statistically significant (log RR = 0.89, 95% CI: −1.23 to 3.01; Appendix 6.1).

## 4 Discussion

We conducted a network meta-analysis of 12 high-quality RCTs evaluating 11 anti-tau therapies for AD. These therapies significantly decreased tau biomarker levels. However, no significant benefits were observed for the primary outcomes (ADCS-Cog, CDR-SB, ADCS-ADL and MMSE) or the tau PET composite SUVR. The SUCRA rankings revealed that MAPT_Rx_ ranked highest in MMSE, with nicotinamide ranked highest in ADAS-Cog. Moreover, gosuranemab ranked highest for t-tau, p-tau, and tau PET composite SUVRs. These results show that anti-tau therapies reduce CSF soluble tau biomarkers, but this does not equate to amelioration of tau pathology, given the lack of effect on tau PET and clinical outcomes, although some drugs focusing on tau synthesis inhibition and posttranslational modification modulation have shown potential efficacy in specific cognitive and daily functions.

AD is a progressive neurodegenerative disease driven by two main mechanisms: the amyloid-β (Aβ) cascade and tau hyperphosphorylation [31]. Tau acts as a key downstream mediator of Aβ pathology, in which its hyperphosphorylation triggers tauopathy, neuronal loss, neuroinflammation, and cognitive decline [32]. Clinical studies have confirmed the correlation between tau pathology and symptom onset in patients with AD [31,33,34]. This evidence highlights the central role of tau in the clinical progression of AD, positioning anti-tau therapies as promising targets.

Influence analysis revealed nicotinamide as a statistical outlier for the assessment of CDR-SB (SDR = −2.66, CD = 0.48). After the outliers were removed, the overall effect sizes increased in CDR-SB (SMD = 0.02 vs. 0.05), but the results remained statistically nonsignificant. In terms of the SUCRA ranking, nicotinamide achieved the highest value in CDR-SB (SUCRA: 98.7) before outlier removal. Nicotinamide, a tau posttranslational modification modulator, may broadly modulate AD pathophysiology, thereby contributing to improvements in cognitive and overall function. MAPT_Rx_ was identified as an outlier for CSF t-tau (SDR = −4.62, CD = 1.05) and p-tau (SDR = −5.94, CD = 1.52). After the outliers were removed, the overall effect sizes were attenuated for t-tau (SMD from −0.97 to −0.69) and p-tau (SMD from −0.98 to −0.48), but the results remained statistically significant, with a moderate effect size. In the SUCRA rankings before outlier removal, MAPT_Rx_ consistently achieved the highest probability of being the most effective intervention for reducing p-tau (SUCRA:100.0). This outlier effect is attributable to the unique upstream mechanism of MAPT_Rx_: as an antisense oligonucleotide, it directly suppresses tau synthesis at the mRNA level, fundamentally reducing tau production [[35], [36], [37], [38]]. The high dose of MAPT_Rx_ and the relatively small sample size (treatment n = 34, placebo n = 12) in this phase Ib trial [2] resulted in a profound and rapid reduction in CSF tau and made it more susceptible to random variability, resulting in an extreme effect estimate.

After excluding outliers, our network meta-analysis revealed significant reductions in CSF tau levels but no significant improvements in the four primary clinical outcomes, namely ADAS-Cog, CDR-SB, ADCS-ADL and MMSE scores, or in the tau PET composite SUVR. These findings are consistent with those of a prior meta-analysis of 34 RCTs by Zheng et al. [39], who reported no significant improvement in ADAS-Cog (MD =−0.77, 95% CI:−1.64 to 0.10), CDR-SB (MD = 0.19, 95% CI:−0.21 to 0.59), ADCS-ADL (MD = 0.48, 95% CI:−1.01 to 1.97), or MMSE (MD = 0.48, 95% CI:−0.19 to 1.14) and nonsignificant changes in CSF t-tau (SMD: −0.08, 95% CI: −0.26 to 0.09) and p-tau (SMD: −0.15, 95% CI: −0.33 to 0.03). The discrepancy in the effects of biomarkers likely stems from differences in the included trials. Our study focused specifically on drugs with well-defined tau-targeting mechanisms, including agents with profound effects on reducing tau, while the previous analysis incorporated a broader range of drug classes, including some nonspecific tau modulators (such as herbal extracts), which may have diluted the overall effect and may not directly have suppressed the overall burden of soluble tau species measured in the CSF. T-tau and p-tau primarily reflect soluble tau species, which may be more readily accessible and responsive to therapeutic clearance. In contrast, tau PET signals capture insoluble, fibrillary tau aggregates that have already formed neurofibrillary tangles [40]. Moreover, the design of most current anti-tau monoclonal antibodies is predicated on the hypothesis that extracellular tau acts as a key vehicle for pathological propagation [41] and that anti-tau monoclonal antibodies act to neutralize extracellular tau seeds to prevent further spread. However, the driving role of extracellular tau in AD progression may be overestimated or not exclusive. It is possible that tau seeds are not accessible to anti-tau monoclonal antibodies if they are spread within exosomes, extracellular vesicles, or between cells [42] rather than being exposed in the interstitial fluid, or potentially, extraneuronal tau spread may simply not be a prominent mechanism through which tau pathology evolves [43]. Therefore, while drugs may clear a substantial proportion of soluble CSF tau, they could miss the protected tau seeds that truly drive pathological propagation, resulting in no significant improvement in the insoluble tau fibrils measured by tau PET. Our results suggest that, compared with dismantling a pre-existing, stable tangle pathology, current anti-tau therapies may be more effective at suppressing new pathologies or clearing soluble species. Although no significant improvements in cognition or function were observed, the present findings provide more concentrated evidence for the clinical evaluation of tau-specific therapies.

In the SUCRA rankings after outlier removal, MAPT_Rx_ ranked highest in MMSE, whereas nicotinamide performed best in ADAS-Cog. Gosuranemab ranked highest in t-tau, p-tau, and tau PET composite SUVR reduction. MAPT_Rx_, the only tau-targeting antisense oligonucleotide included in this analysis, was associated with the greatest improvement in the MMSE score. By directly degrading MAPT mRNA, MAPT_Rx_ reduces tau protein synthesis at its source, potentially providing more fundamental suppression of tau pathology. This early, preemptive intervention may preserve neuronal and synaptic function more effectively, which could explain its favorable effect on global cognitive function as measured by MMSE. Nicotinamide ranked first in ADAS-Cog, likely because of improved neuronal energy metabolism, enhanced autophagy, and the counteraction of tau acetylation [44]. These results are relatively consistent with the subgroup analyses in the study by Zheng et al. [39]. Although nicotinamide was not included in the previous meta-analysis, tau posttranslational modifications significantly improved the ADAS-Cog (MD: −0.80, 95% CI: −1.43 to −0.17) in the subgroup analysis [39]. As a monoclonal antibody targeting extracellular tau, gosuranemab enhances the clearance of soluble tau species [45,46], explaining its pronounced effects on both fluid biomarkers and PET-measured fibrillary tau. In contrast, tau aggregation inhibitors primarily target intracellular neurofibrillary tangles with a limited effect on soluble tau, whereas phosphorylation modulators act upstream and are less effective at clearing existing pathological tau. Furthermore, the optimized dosing regimen in the gosuranemab trial ensured high drug exposure, maximizing its biological efficacy and performance in indirect comparisons [30].

The influence analysis for safety analyses revealed gosuranemab (SDR = −1.98, CD = 5.47) as an outlier for injection-site reactions. After the outliers were removed, the risk of injection-site reactions changed from nonsignificant to significant. In the gosuranemab study [30], the incidence of injection-site reactions was unusually low, which may be related to the injection technique, site care, or adverse event recording methods used in the study, causing gosuranemab to act as a negative outlier in the pooled analysis. After this study was excluded, the risk of injection-site reactions became significant, indicating that such adverse reactions are common among other anti-tau drugs, particularly monoclonal antibodies, which may be attributed to the large molecular nature and local immunostimulatory properties of these drugs, consistent with findings from clinical studies of monoclonal antibodies in other fields [47]. Safety findings have suggested an acceptable overall safety profile and support the feasibility of anti-tau therapies in AD patients. Nonetheless, monitoring injection-site reaction risks is essential during treatment.

Meta-regression revealed an association between t-tau reduction and age, suggesting that younger patients might experience greater t-tau reductions with anti-tau therapy. However, this association became nonsignificant after the exclusion of the outlier of the MAPT_Rx_ study. This shift is highly indicative of a confounding effect driven by the MAPT_Rx_ study cohort. Compared with both the general AD population and other trials included in this meta-analysis, the MAPT_Rx_ trial enrolled a notably younger population (mean age 66 years old) with a disproportionately high percentage of early-onset AD participants (46%) [2]. Crucially, owing to the potent upstream mechanism of action of MAPT_Rx_, this trial demonstrated the most profound reduction in CSF t-tau levels among all the studies. Therefore, in the regression model, MAPT_Rx_ created a statistically influential point that artificially pulled the regression line, generating a spurious association according to which younger age resulted in greater t-tau reduction.

The sensitivity analysis revealed that semorinemab substantially influenced the overall estimates for injection-site reactions. Its exclusion led to fundamental shifts in the statistical significance of the results. As a monoclonal antibody, the inherent immunogenicity and local irritability of semorinemab led to a higher incidence of injection-site reactions. However, only three studies contributed to the analysis of injection-site reactions, and this safety result should be interpreted cautiously.

Several methodological and biological factors may influence the interpretation of our findings. First, the statistical power to detect clinical efficacy varied widely across the included studies, as most were early‑phase (Phase Ib/II) exploratory trials focused on target engagement, tolerability, safety, and biomarker changes rather than cognitive or functional endpoints. Second, anti-tau agents target distinct tau regions, epitopes, or pathological conformations. For example, some monoclonal antibodies bind to the amino-terminus, mid-domain or carboxy-terminus of tau, whereas others target specific phosphorylation sites or aggregate forms; antisense oligonucleotides suppress total tau synthesis, and small molecules modulate post-translational modifications or inhibit aggregation. These mechanistic differences may lead to divergent effects on soluble versus insoluble tau species and on extracellular seed propagation versus intracellular tangle formation, potentially explaining the heterogeneous biomarkers and clinical responses observed. Third, the trial durations ranged from 10 to 104 weeks, which may be insufficient to translate sustained reductions in soluble tau biomarkers into measurable clinical benefits, given the slowly progressive nature of tau pathology. Finally, baseline disease severity and biomarker levels, including CSF tau concentrations and tau PET SUVR, varied across study populations, and patients at earlier stages might show greater responsiveness than those at advanced stages. Collectively, these factors underscore the need for adequately powered, longer-duration phase III trials with optimized patient selection and sensitive outcome measures to fully evaluate the clinical potential of anti-tau therapies.

Our meta-analysis has several limitations. First, the strict inclusion criteria resulted in a limited number of eligible studies, and missing data from some included RCTs could not be retrieved, which together restrict robust conclusions. Second, this research was confined to PubMed, the Cochrane Library, and Embase; studies published after December 2025, trial registries, and gray literature were excluded, thereby increasing the risk of publication bias from unreported results. Third, although the risk of bias was assessed with the standard Cochrane Risk of Bias Tool for Randomized Trials (RoB 2), it still might have undercaptured Alzheimer’s-specific issues (e.g., tau PET variability); specialized tools, such as RoB-Dem, could improve future assessments. Fourth, the method of combining data across both different dosage groups of treatment and different detection regions of tau PET may have weakened the dose‒response relationship or the specific tau clearance effect of the drug on brain regions vulnerable to early AD (e.g., the temporal lobe), thus potentially obscuring the true therapeutic efficacy in our analysis. Fifth, we focused on predefined primary outcomes (ADAS-Cog, CDR-SB, ADCS-ADL, and MMSE), potentially missing benefits captured by alternative validated scales, such as composite scales. Furthermore, transitivity in the network meta-analysis was evaluated conservatively, which may have inaccurately ranked drug efficacy because of unaddressed trial heterogeneity. Additionally, the meta-regression was limited to baseline MMSE score, CDR-SB score, age, sex, and tau PET composite SUVR, while omitting key moderators, such as APOE ε4 carrier status, which might delineate responsive subgroups. Moreover, the small number of studies available for meta-regression reduced the statistical power and reliability of the estimates, and without covariate-adjusted meta-regression, heterogeneities were not fully mitigated. Finally, we did not perform subgroup analyses or explore dose‒response relationships, which could have provided insights into varying treatment effects across patient characteristics or dosing regimens.

Notably, several new anti-tau drugs under development (such as etalanetug and MK-2214) were not included in this study because they did not meet our eligibility criteria, in part because phase II clinical trials have not yet been completed. Nevertheless, these agents may still hold therapeutic potential and warrant evaluation in future updates once later-phase clinical data become available.

## 5 Conclusions

This study demonstrated that anti-tau agents effectively reduced p-tau and t-tau levels in the CSF of patients with AD, but this biological improvement did not achieve clinical benefits in terms of cognitive and global functions. However, several new anti-tau drugs under development, such as etalanetug and MK-2214, may still hold therapeutic potential, but they were excluded because of their disqualifying early-stage status (e.g., being in phase I or incomplete phase II trials). Among all the included trials, SUCRA rankings indicated that nicotinamide ranked highest for ADAS-Cog(71.4%), and MAPT_Rx_ ranked highest for MMSE (93.5%), suggesting that specific anti-tau approaches may have a cognitive improvement effect and/or disease-modifying potential that merits confirmation in further trials. In terms of adverse events, anti-tau therapy demonstrated favorable overall tolerability, in addition to an increased risk of injection-site reactions. Given the consistent failure of phase II/III trials to show clinical efficacy, the main value of this meta-analysis is to define the evidence boundary for future studies. Future development of anti-tau therapies should prioritize the depth and speed of pathological tau clearance, establish efficacy thresholds for tau reduction, and advance effective combination strategies, including combinations with anti-amyloid therapies or neuroprotective agents. Although overall clinical efficacy was not reported in this meta-analysis, taken together, the consistent beneficial effects on biomarkers and good safety support the continued investigation of anti-tau therapies, particularly those that have shown potential benefits on certain cognitive or functional measures in their completed clinical trials.

## Ethics approval and consent to participate

Not applicable.

## Consent statement

Not applicable.

## Funding

This work was supported by the Science and Technology Innovation 2030—Major Project (grant number 2021ZD0201805) and the Tianjin Key Medical Discipline (Specialty) Construction Project (grant number TJYXZDXK-004A).

## Declaration of generative AI and AI-assisted technologies in the writing process

I have not used any AI at all.

## CRediT authorship contribution statement

**Yuetao Hu:** Writing – original draft, Conceptualization. **Yuning Cheng:** Formal analysis, Data curation. **Yanqu Su:** Writing – original draft, Data curation. **Zeming Han:** Writing – original draft, Visualization. **Xiaohan Shen:** Investigation. **Hantian Hu:** Investigation. **Qi Wang:** Project administration. **Siqi Wan:** Resources. **Hanyu Li:** Software. **Nan Zhang:** Writing – review & editing, Funding acquisition, Conceptualization.

## Declaration of competing interest

The authors have no conflicts of interest to declare.

## Data Availability

The data that support the findings of this study are available on request from the corresponding author. The data is not publicly available due to privacy or ethical restrictions.
