# Supplementary material for: Efficacy and safety of anti-tau drugs for Alzheimer’s disease: A systematic review and network meta-analysis
Source: J Prev Alzheimers Dis. 2026 Nov 7;13(10):100693. doi: 10.1016/j.tjpad.2026.100693 (PMC13761260; doi:10.1016/j.tjpad.2026.100693)
Supplement: Supplementary file 1 [file mmc1.docx]

**Supplement appendix**

Supplement to:

**Efficacy and Safety of Anti-Tau Drugs for Alzheimer’s Disease: A Systematic Review and Network Meta-Analysis**

[Appendix 1 Risk of bias assessments	4](#_Toc32406)

[1.1 The risk of bias summary assessment using the Risk of Bias tool.	4](#_Toc28980)

[1.2 The risk of bias summary of the included studies.	5](#_Toc20906)

[Appendix 2 Influence statistics	6](#_Toc15620)

[2.1 Results of the influence statistics as the basis for outlier identification for the primary outcomes.	6](#_Toc29621)

[2.2 Results of the influence statistics as the basis for outlier identification for the secondary outcomes.	8](#_Toc18582)

[2.3 Results of the influence statistics as the basis for outlier identification for adverse events.	9](#_Toc30090)

[Appendix 3 Results of meta-analysis	10](#_Toc26777)

[3.1 Forest plots after outlier removal	10](#_Toc27550)

[3.1.1 Forest plots of the primary outcomes after outlier removal.	10](#_Toc12324)

[3.1.2 Forest plots of the secondary outcomes after outlier removal.	11](#_Toc4924)

[3.2 Forest plots without outlier removal	12](#_Toc19370)

[3.2.1 Forest plots of the primary outcomes without outlier removal.	12](#_Toc25139)

[3.2.2 Forest plots of the secondary outcomes without outlier removal.	13](#_Toc28752)

[3.2.3 Forest plots of meta-analyses of adverse events without outlier removal.	14](#_Toc23871)

[3.3 Pooled estimations of clinical effects with additional Begg’s test and Egger’s test without excluding the outliers.	15](#_Toc27470)

[Appendix 4 Results of network meta-analysis	17](#_Toc12926)

[4.1 Network meta-analysis without outlier removal	17](#_Toc30978)

[4.1.1 Network meta-analysis of the primary outcomes without outlier removal.	17](#_Toc16551)

[4.1.2 Network meta-analysis of the secondary outcomes without outlier removal.	18](#_Toc2518)

[4.2 SUCRA values for all outcomes under different interventions without excluding the outliers.	20](#_Toc14560)

[Appendix 5 Meta-regression	21](#_Toc2864)

[5.1 Meta-regression after outlier removal.	21](#_Toc14438)

[5.1.1 Meta-regression of the primary outcomes after outlier removal.	21](#_Toc15617)

[5.1.2 Meta-regression of the secondary outcomes after outlier removal.	23](#_Toc24683)

[5.1.3 Meta-regression of the primary outcomes on biomarker changes	25](#_Toc8367)

[5.2 Meta-regression without outlier removal.	26](#_Toc26059)

[5.2.1 Meta-regression of the primary outcomes without outlier removal.	26](#_Toc13423)

[5.2.2 Meta-regression of the secondary outcomes without outlier removal.	28](#_Toc8076)

[5.2.3 Meta-regression of the primary outcomes on t-tau changes without outlier removal. The figure presents a meta-regression of cognitive outcomes (CDR-SB and MMSE) on biomarker changes (a reduction in t-tau).	29](#_Toc1609)

[5.2.4 Meta-regression of the primary outcomes on p-tau changes without outlier removal. The figure presents a meta-regression of cognitive outcomes (CDR-SB and MMSE) on biomarker changes (a reduction in p-tau).	30](#_Toc4680)

[Appendix 6 Sensitivity analysis	31](#_Toc17496)

[6.1 Sensitivity analysis after outlier removal.	31](#_Toc10221)

[6.1.1 Forest plots of sensitivity analysis of the primary outcomes and secondary outcomes after outlier removal.	31](#_Toc23567)

[6.1.2 Forest plots of the sensitivity analysis of adverse events after outlier removal.	32](#_Toc32376)

[6.2 Sensitivity analysis without outlier removal.	33](#_Toc2174)

[6.2.1 Forest plots of the sensitivity analysis of the primary outcomes and secondary outcomes without outlier removal.	33](#_Toc19865)

[6.2.2 Forest plots of the sensitivity analysis of adverse events without outlier removal.	34](#_Toc28656)

# Appendix 1 Risk of bias assessments

## 1.1 The risk of bias summary assessment using the Risk of Bias tool.

## 1.2 The risk of bias summary of the included studies.

# Appendix 2 Influence statistics

2.1 Results of the influence statistics as the basis for outlier identification for the primary outcomes. Influence analysis revealed the outliers of the study in ADAS-Cog (A), CDR-SB (B), ADCS-ADL (C), and MMSE (D). The outliers that were identified by the influence analysis are marked by a circle, with absolute SDR values exceeding 1.96 and CD values exceeding 0.45.

Abbreviations: ADAS-Cog, Alzheimer’s Disease Assessment Scale - Cognitive Subscale; CDR-SB, Clinical Dementia Rating - Sum of Boxes; ADCS-ADL, Alzheimer’s Disease Cooperative Study - Activities of Daily Living; MMSE, Mini Mental State Examination; SDR, studentized deleted residuals; CD, Cook’s distance.

2.2 Results of the influence statistics as the basis for outlier identification for the secondary outcomes. Influence analysis revealed the outliers of the study in t-Tau (A), p-Tau (B) and tau PET (C). The outliers that were identified by the influence analysis are marked by a circle, with absolute SDR values exceeding 1.96 and CD values exceeding 0.45.

Abbreviations: t-tau, total tau protein in the cerebrospinal fluid; p-tau, phosphorylated tau protein in the cerebrospinal fluid; PET, positron emission tomography; SUVR, standardized uptake value ratio; SDR, studentized deleted residuals; CD, Cook’s distance.

2.3 Results of the influence statistics as the basis for outlier identification for adverse events. Influence analysis revealed the outliers of the study in fatigue (A), nausea (B), diarrhea (C), dizziness (D), fall (E), urinary tract infection (F), and injection site reaction (G). The outliers that were identified by the influence analysis are marked by a circle, with absolute SDR values exceeding 1.96 and CD values exceeding 0.45.

Abbreviations: CI, confidence interval; SDR, studentized deleted residuals; CD, Cook’s distance.

# Appendix 3 Results of meta-analysis

## 3.1 Forest plots after outlier removal

3.1.1 Forest plots of the primary outcomes after outlier removal. Forest plots depict meta-analysis results for anti-tau drug efficacy in ADAS-Cog (A), CDR-SB (B), ADCS-ADL (C), and MMSE (D). Effects to the left of the line (no effect, at 0) favor treatment for A and B, whereas those to the right favor treatment for C and D because lower scores indicate better performance in ADAS-Cog and CDR-SB, along with worse performance in ADCS-ADL and MMSE.

3.1.2 Forest plots of the secondary outcomes after outlier removal. Forest plots depict meta-analysis results for anti-tau drug efficacy in t-tau (A), p-tau (B) and tau PET (C).

Abbreviations: t-tau, total tau protein in the cerebrospinal fluid; p-tau, phosphorylated tau protein in the cerebrospinal fluid; PET, positron emission tomography; SUVR, standardized uptake value ratio.

## 3.2 Forest plots without outlier removal

3.2.1 Forest plots of the primary outcomes without outlier removal. Forest plots depict meta-analysis results for anti-tau drug efficacy in CDR-SB. No outliers were identified in the other primary outcomes (the results are presented in Appendix 3.1.1).

Abbreviations: CDR-SB, Clinical Dementia Rating - Sum of Boxes.

3.2.2 Forest plots of the secondary outcomes without outlier removal. Forest plots depict meta-analysis results for the efficacy of anti-tau drugs in CSF t-tau (A) and p-tau (B). No outliers were identified in the other secondary outcomes (the results are presented in Appendix 3.1.2).

Abbreviations: t-tau, total tau; p-tau, phosphorylated tau.

3.2.3 Forest plots of meta-analyses of adverse events without outlier removal. Effect sizes are presented as relative ratios with 95% confidence intervals (CIs). Forest plots depict meta-analysis results for adverse events of anti-tau drugs, including injection site reactions (A). No outliers were identified in the other adverse events (the results are presented in the main text of Figure 4).

Abbreviations: CI, confidence interval.

## 3.3 Pooled estimations of clinical effects with additional Begg’s test and Egger’s test without excluding the outliers.

| **Outcomes** | **SMD [95%CI]** | **τ^2^** | **P value** | **I^2^** | **H^2^** | **df** | **λ^2^** | **z** | **P for Cochran’s Q test** | **Begg’s test Kendall’s score** | **P for Begg’s test** | **Egger’s test t value** | **P for Egger’s test** |
| --- | --- | --- | --- | --- | --- | --- | --- | --- | --- | --- | --- | --- | --- |
| **ADAS-Cog** | -0.01[-0.08, 0.10] | - | 0.850 | 43.80% | 1.78 | 9 | 16.01 | 0.19 | 0.067 | -13.00 | 0.245 | -1.74 | 0.119 |
| **CDR** | -0.02[-0.08,0.12] | - | 0.700 | 21.20% | 1.27 | 6 | 7.62 | 0.39 | 0.267 | -13.00 | 0.072 | -1.79 | 0.134 |
| **ADCS-ADL** | -0.09[-0.21,0.02] | - | 0.122 | 1.80% | 1.02 | 4 | 4.07 | -1.55 | 0.396 | -6.00 | 0.221 | -2.02 | 0.136 |
| **MMSE** | -0.04[-0.14,0.07] | - | 0.495 | 17.40% | 1.21 | 8 | 9.69 | -0.68 | 0.287 | 18.00 | 0.076 | 2.22 | 0.062 |
| **t-tau** | -0.97[-1.62, -0.31] | 0.54 | 0.004 | 84.40% | 6.40 | 5 | 31.98 | -2.90 | ＜0.01 | -3.00 | 0.707 | -0.50 | 0.641 |
| **p-tau** | -0.98[-1.78, -0.18] | 0.72 | 0.016 | 88.80% | 8.99 | 4 | 35.71 | -2.40 | ＜0.01 | -4.00 | 0.46 | -1.32 | 0.280 |
| **tau PET SUVR** | 0.01[-0.12, 0.15] | - | 0.862 | 0.00% | 1.00 | 3 | 2.99 | -2.19 | 0.393 | 4.00 | 0.308 | 2.26 | 0.152 |

Note: The **τ^2^** value is only reported for analyses using a random-effects model (I² ≥ 50%), and it is not applicable under the fixed-effects model (I² < 50%).

Abbreviations: ADAS-Cog, Alzheimer’s Disease Assessment Scale - Cognitive Subscale; CDR-SB, Clinical Dementia Rating - Sum of Boxes; ADCS-ADL, Alzheimer’s Disease Cooperative Study - Activities of Daily Living; MMSE, Mini Mental State Examination; t-tau, total tau protein in the cerebrospinal fluid; p-tau, phosphorylated tau protein in the cerebrospinal fluid; PET, positron emission tomography; SUVR, standardized uptake value ratio.

# Appendix 4 Results of network meta-analysis

## 4.1 Network meta-analysis without outlier removal

### 4.1.1 Network meta-analysis of the primary outcomes without outlier removal.

The figure presents three plots for CDR-SB. The network plot (A) illustrates the comparisons among the interventions, wherein node size is proportional to the number of participants, and edge thickness reflects the sample size for direct comparisons. The SUCRA ranking plot (B) illustrates the probability of each intervention being the best treatment. The forest plot (C) displays network effect sizes versus placebo with 95% credible intervals. No outliers were identified in the other primary outcomes (the results are presented in the main text of Figure 2).

Abbreviations: CDR-SB, Clinical Dementia Rating - Sum of Boxes; SUCRA, surface under the cumulative ranking curve.

### 4.1.2 Network meta-analysis of the secondary outcomes without outlier removal.

For each biomarker outcome, including t-Tau (A-C) and p-Tau (D-F), the figure presents three plots: (A, D) network plots show intervention comparisons, with node size reflecting participant numbers and edge thickness indicating sample sizes for the direct comparisons; (B, E) SUCRA ranking plots illustrate the probability of each intervention being the best treatment; and (C, F) forest plots display network effect sizes versus placebo with 95% credible intervals. No outliers were identified in the other secondary outcomes (the results are presented in the main text of Figure 3).

Abbreviations: t-tau, total tau protein in the cerebrospinal fluid; p-tau, phosphorylated tau protein in the cerebrospinal fluid.

## 4.2 SUCRA values for all outcomes under different interventions without excluding the outliers.

| Intervention | ADAS-Cog | ADCS-ADL | CDR-SB | MMSE | T-tau | P-tau | Tau PET SUVR |
| --- | --- | --- | --- | --- | --- | --- | --- |
| MT | 58.4 | - | - | - | - | - | - |
| Tilavonemab | 49.3 | - | 47.7 | 41.4 | - | - | - |
| Semorinemab | 61.8 | 64.2 | 29.6 | 86.0 | 53.2 | 48.6 | 48.2 |
| Gosuranemab | 26.3 | 50.8 | 46.3 | 22.3 | 92.7 | 77.0 | 87.1 |
| VEL015 | 21.6 | - | - | 48.7 | 43.0 | 27.0 | - |
| Tideglusib | 50.0 | 48.1 | - | 39.2 | - | - | - |
| Nicotinamide | 71.4 | 7.4 | 98.7 | - | 48.9 | 38.7 | - |
| Lithium | 65.3 | - | - | 26.0 | - | - | - |
| AADvac1 | - | - | 53.2 | 32.4 | 64.8 | - | - |
| Zagotenemab | - | - | 40.0 | 58.4 | - | - | 10.4 |
| MAPTRx | - | - | - | 93.5 | 39.1 | 100.0 | - |

Abbreviations: CDR-SB, Clinical Dementia Rating - Sum of Boxes; t-tau, total tau protein in the cerebrospinal fluid; p-tau, phosphorylated tau protein in the cerebrospinal fluid.

# Appendix 5 Meta-regression

## 5.1 Meta-regression after outlier removal.

5.1.1 Meta-regression of the primary outcomes after outlier removal. Plots are used to examine associations between the primary outcomes (including ADAS-Cog, CDR-SB, ADCS-ADL, and MMSE) and baseline factors. Dark blue dots represent outcomes associated with age at baseline; light blue dots represent outcomes associated with the proportion of females at baseline; light green dots represent outcomes associated with the CDR at baseline; red dots represent outcomes associated with the MMSE at baseline; yellow dots represent outcomes associated with the t-tau level at baseline; and dark green dots represent outcomes associated with the tau PET composite SUVR at baseline. The vertical arrows indicate the direction of the outcome improvement.

Abbreviations: ADAS-Cog, Alzheimer’s Disease Assessment Scale - Cognitive Subscale; CDR-SB, Clinical Dementia Rating - Sum of Boxes; ADCS-ADL, Alzheimer’s Disease Cooperative Study - Activities of Daily Living; MMSE, Mini Mental State Examination.

5.1.2 Meta-regression of the secondary outcomes after outlier removal. Plots are used to examine associations between biomarker changes (including t-tau, p-tau, and the tau PET composite SUVR) and baseline factors. Dark blue dots represent outcomes associated with age at baseline; light blue dots represent outcomes associated with the proportion of females at baseline; green dots represent outcomes associated with the CDR at baseline; red dots represent outcomes associated with the MMSE at baseline; and yellow dots represent outcomes associated with the t-tau level at baseline. The vertical arrows indicate the direction of outcome improvement.

Abbreviations: CSF, cerebrospinal fluid; t-tau, total tau; p-tau, phosphorylated tau; MMSE, Mini Mental State Examination; PET, positron emission tomography; SUVR, standardized uptake value ratio.

5.1.3 Meta-regression of the primary outcomes on biomarker changes after outlier removal. Plots are used to examine associations between primary outcomes (including ADAS-Cog, CDR-SB, ADCS-ADL, and MMSE) and the extent of tau reduction measured with PET and CSF biomarkers. Green dots represent outcomes associated with a reduction in t-tau; blue dots represent outcomes associated with a reduction in p-tau; and purple dots represent outcomes associated with a reduction in the tau PET composite SUVR. The vertical arrows indicate the direction of outcome improvement.

Abbreviations: ADAS-Cog, Alzheimer’s Disease Assessment Scale - Cognitive Subscale; CDR-SB, Clinical Dementia Rating - Sum of Boxes; ADCS-ADL, Alzheimer’s Disease Cooperative Study - Activities of Daily Living; MMSE, Mini Mental State Examination; CSF, cerebrospinal fluid; t-Tau, total tau; p-tau, phosphorylated tau; PET, positron emission tomography; SUVR, standardized uptake value ratio.

## 5.2 Meta-regression without outlier removal.

5.2.1 Meta-regression of the primary outcomes without outlier removal. Plots are used to examine associations between the primary outcomes of CDR-SB and baseline factors. Dark blue dots represent outcomes associated with age at baseline; light blue dots represent outcomes associated with the proportion of females at baseline; light green dots represent outcomes associated with the CDR at baseline; red dots represent outcomes associated with the MMSE at baseline; and yellow dots represent outcomes associated with the t-tau level at baseline. The vertical arrows indicate the direction of outcome improvement. No outliers were identified in the other primary outcomes (the results are presented in Appendix 7.1.1).

5.2.2 Meta-regression of the secondary outcomes without outlier removal. Plots are used to examine associations between biomarker changes (t-tau and p-tau) and baseline factors. Dark blue dots represent outcomes associated with age at baseline; light blue dots represent outcomes associated with the proportion of females at baseline; light green dots represent outcomes associated with the CDR at baseline; red dots represent outcomes associated with the MMSE at baseline; and yellow dots represent outcomes associated with the t-tau level at baseline. The vertical arrows indicate the direction of outcome improvement. No outliers were identified in the other secondary outcomes (the results are presented in Appendix 7.1.2).

Abbreviations: CSF, cerebrospinal fluid; t-tau, total tau; p-tau, phosphorylated tau; MMSE, Mini Mental State Examination; PET, positron emission tomography; SUVR, standardized uptake value ratio.

5.2.3 Meta-regression of the primary outcomes on t-tau changes without outlier removal. The figure presents a meta-regression of cognitive outcomes (CDR-SB and MMSE) on biomarker changes (a reduction in t-tau). No outliers were identified in the other secondary outcomes (the results are presented in Appendix 7.1.3).

Abbreviations: CDR-SB, Clinical Dementia Rating - Sum of Boxes; MMSE, Mini Mental State Examination; CSF, cerebrospinal fluid; t-tau, total tau.

5.2.4 Meta-regression of the primary outcomes on p-tau changes without outlier removal. The figure presents a meta-regression of cognitive outcomes (CDR-SB and MMSE) on biomarker changes (a reduction in p-tau). No outliers were identified in the other secondary outcomes (the results are presented in Appendix 7.1.3). Although influence analysis identified outliers for the CDR-SB and MMSE, meta-regression was not feasible because of insufficient data after outlier removal (<3 study arms).

Abbreviations: CDR-SB, Clinical Dementia Rating - Sum of Boxes; MMSE, Mini Mental State Examination; CSF, cerebrospinal fluid; t-tau, total tau.

# Appendix 6 Sensitivity analysis

## 6.1 Sensitivity analysis after outlier removal.

6.1.1 Forest plots of sensitivity analysis of the primary outcomes and secondary outcomes after outlier removal. Sensitivity analysis of ADAS-Cog (A), CDR-SB (B), ADCS-ADL (C), MMSE (D), t-tau (E), p-tau (F) and tau PET composite SUVR (G) using the sequential exclusion method by excluding each study and recalculating the effect size with the remaining studies. In each plot, the dots represent the effect estimates after excluding a particular study, with the horizontal lines indicating the corresponding 95% CIs, wherein a result is considered to be robust if none of the CIs included the zero point. Conversely, if the omission of any single study leads to a CI including the zero point, the result is considered to not be robust, thus indicating that the omitted study substantially influences the overall conclusion.

Abbreviations: ADAS-Cog, Alzheimer’s Disease Assessment Scale - Cognitive Subscale; CDR-SB, Clinical Dementia Rating - Sum of Boxes; ADCS-ADL, Alzheimer’s Disease Cooperative Study - Activities of Daily Living; MMSE, Mini Mental State Examination; t-Tau, total tau protein in the cerebrospinal fluid; p-Tau, phosphorylated tau protein in the cerebrospinal fluid; PET, positron emission tomography; SUVR, standardized uptake value ratio.

6.1.2 Forest plots of the sensitivity analysis of adverse events after outlier removal. Sensitivity analysis of fatigue (A), nausea (B), diarrhea (C), dizziness (D), fall (E), urinary tract infection (F), and injection site reaction (G) using the sequential exclusion method by excluding each study and recalculating the effect size with the remaining studies. In each plot, the dots represent the effect estimates after excluding a particular study, with the horizontal lines indicating the corresponding 95% CIs, wherein a result is considered to be robust if none of the CIs included the zero point. Conversely, if the omission of any single study leads to a CI including the zero point, the result is considered to not be robust, thus indicating that the omitted study substantially influences the overall conclusion.

Abbreviations: CI, confidence interval.

## 6.2 Sensitivity analysis without outlier removal.

6.2.1 Forest plots of the sensitivity analysis of the primary outcomes and secondary outcomes without outlier removal. Sensitivity analysis of CDR-SB (A), t-tau (B) and p-tau (C) by excluding each study and recalculating the effect size with the remaining studies. In each plot, the dots represent the effect estimates after excluding a particular study, with the horizontal lines indicating the corresponding 95% CIs, wherein a result is considered to be robust if none of the CIs included the zero point. Conversely, if the omission of any single study leads to a CI including the zero point, the result is considered to not be robust, thus indicating that the omitted study substantially influences the overall conclusion. No outliers were identified in the other efficacy results (the results are presented in Appendix 8.1.1).

Abbreviations: ADAS-Cog, Alzheimer’s Disease Assessment Scale - Cognitive Subscale; CDR-SB, Clinical Dementia Rating - Sum of Boxes; ADCS-ADL, Alzheimer’s Disease Cooperative Study - Activities of Daily Living; MMSE, Mini Mental State Examination; t-tau, total tau protein in the cerebrospinal fluid; p-tau, phosphorylated tau protein in the cerebrospinal fluid; PET, positron emission tomography; SUVR, standardized uptake value ratio; CI, confidence interval.

6.2.2 Forest plots of the sensitivity analysis of adverse events without outlier removal. Sensitivity analysis of the injection site reaction by excluding each study and recalculating the effect size with the remaining studies. In each plot, the dots represent the effect estimates after excluding a particular study, with the horizontal lines indicating the corresponding 95% CIs, wherein a result is considered to be robust if none of the CIs included the zero point. Conversely, if the omission of any single study leads to a CI including the zero point, the result is considered to not be robust, thus indicating that the omitted study substantially influences the overall conclusion. No outliers were identified in the other efficacy results (the results are presented in Appendix 8.1.2).

Abbreviations: CI, confidence interval.
